# Severe Metabolic Acidosis: A Case of Triple Hit with Ketogenic Diet, Vinegar, and Metformin in an Obese Patient

**DOI:** 10.1155/2020/8861978

**Published:** 2020-09-15

**Authors:** Abdul Mughees Rana, Kannan Mansoor, Salman Assad, Mahmoud Abouzid, Iheanyichukwu Ogu, Ghassan Salim Issa Bandak

**Affiliations:** ^1^Department of Internal Medicine University Huntington, 1249 15th Street Suite 2000, Huntington, WV 25514, USA; ^2^Department of Neurology Marshall University Joan C. Edwards School of Medicine, 1600 Medical Center Drive Office B561, Huntington, WV, USA

## Abstract

Obesity is an epidemic with high burden of disease. It is directly proportional to increased risk of morbidity and mortality. Ketogenic diet and herbal supplements have recently gained popularity amongst patients struggling with weight loss. There are limited data available for most of these supplements contrary to the claims laid by the mainstream media. Due to lack of awareness, this patient population is at high risk of adverse effects. We present a case of severe acidosis secondary to ketogenic diet and acetic acid (vinegar) ingestion. The use of dietary acetic acid is usually well tolerated; however, in this case, the large quantities and presence of acute renal injury and metformin use may have worsened the acidosis. Severe ketosis in setting of ketogenic diets is a serious complication, which is infrequently reported in literature. Many of these diets and supplements may seem harmless, but as our case illustrates, when combined with other risk factors, patients can face serious adverse effects and even require hospitalization. It is imperative that such dietary practices are physician supervised to avoid complications. With the recent surge of over-the-counter weight loss supplements and ketogenic diets, physicians also need to engage in dietary discussion with patients when attempting to lose weight.

## 1. Introduction

Obesity is an epidemic with high burden of disease. In 2016, the prevalence of obesity among adults in the United States was close to 40% [1]. Obesity is related to worse outcomes and increased physical morbidity as well as mental health disease [2]. Weight loss is a challenging journey. More than 50–60% of the American adult obese population embarks on this journey yearly [3, 4]. Obese patients try different methods and strategies to lose weight including dietary modifications, dietary restrictions, exercise, weight loss medications, or programs [3, 4]. Low carbohydrate, or ketogenic, diets have gained popularity over the past few years. Ketogenic diets and reducing carbohydrate intake will lead to weight loss through different mechanisms including appetite suppression, increased lipolysis, and changes in lipid metabolism as well as gluconeogenesis [5, 6]. Acetic acid ingestion has also been proposed to be beneficial in weight management [7]. Obese patients seek medical information and help with weight management from various sources including television, online, and social media platforms [8]. These sources may provide unreliable information [8–10]. This may translate into patients engaging in new dietary habits without consulting medical professionals. We present here a unique case of a young female patient who presented to our hospital with severe acidosis secondary to ketogenic diet and acetic acid ingestion. To our knowledge, this is the first case of combined ketogenic acid, metformin use, and acetic acid ingestion leading to severe acidemia in literature.

## 2. Case Presentation

A 43-year-old female with a medical history significant for diabetes mellitus type 2, hypertension, iron deficiency anemia, gastroesophageal reflux disease, and Potts syndrome presented to the emergency department with complaints of weakness and lightheadedness for two weeks. The patient started developing intermittent nausea without any abdominal pain, vomiting, or diarrhea. Symptoms started two weeks prior to presentation. The patient reported no fever, no chills, denied urinary urgency, or increased frequency but complained of dysuria. She also denied any chest pain, cough, or dyspnea. She reported recent weight loss of approximately 14 lbs after starting on ketogenic diet along with regular ingestion of apple cid vinegar. She would drink it as a stand-alone drink or add it to various foods in the last two months. Her diet included little to no carbohydrate. She did not report that she sought advice from a medical professional prior to initiation of her diet. She denied illicit drug use, significant alcohol intake, or ingestion of locally produced alcohols such as moonshine. She consumed alcohol on an infrequent basis, and her last drink was a half glass of wine a day prior to presentation. She denied any prior episodes of diabetic ketoacidosis or use of sodium-glucose cotransporter-2 (SGLT2) inhibitors. She admitted to taking ibuprofen or acetaminophen occasionally for pain. Her medications included lisinopril, hydrochlorothiazide, ferrous sulphate, omeprazole, gabapentin, and metformin. The dose of metformin was increased from 500 mg twice daily to 2000 twice daily 4–5 months before admission. On arrival to the emergency department, she was found to have hypotension and tachycardia. Physical exam was unremarkable except for labored breathing and dry mucous membranes. Her labs revealed metabolic acidosis (pH 7.15) with an increased anion gap of 27 mEq/L, lactic acid of 4.9 mmol/L, blood sugar of 108 mg/dl, serum ethanol level of 18 mg/dL, and elevated creatinine level of 2.7 mg/dL (Table 1). She received 3 L intravenous bolus of normal saline (0.9% NaCl) and was started on IV Dextrose 5% in water (D5W) @100 ml/hr for suspicion of starvation ketosis. She also received 100 mEq of sodium bicarbonate intravenously in addition to initiation of fomepizole empirically, given an elevated osmolal gap of 18 mOsm/kg. With volume expansion, bicarbonate supplementation, as well as dextrose supplementation, the patient's acidosis, acute kidney injury (AKI), and ketosis resolved. Nephrology service was consulted to assist in patient's care, and after reviewing case, they agreed that her acidosis was likely a combination of starvation ketosis, in setting of acetic acid consumption, hypovolemia, and AKI. Other etiologies like ingestion of methanol, isopropyl alcohol, salicylate, or D lactic acidosis were also ruled out. She denied frequent use of acetaminophen; hence, diagnosis of pyroglutamic aciduria was not entertained. Lactic acid level returned to normal within 4 hours and her anion gap closed in the first 14 hours after presentation. A repeat venous pH was recorded as 7.35. On urinalysis, the patient was positive for a urinary tract infection, and the patient endorsed that she had dysuria. The patient was started on IV ceftriaxone. On day 2, the patient's lab work-up was within normal limits. The patient was counseled about dietary intake and was told to abstain from use of vinegar. Metformin was discontinued. The patient was discharged with an endocrinology outpatient follow-up. Laboratory investigations at time of admission and discharge are outlined in Table 1.

## 3. Discussion

Ketogenic diets are popular among patients seeking weight loss. However, there is no clear consensus on the overall health benefits of these diets [11, 12]. During states of low carbohydrate intake, body will promote gluconeogenesis for endogenous glucose production. After few days of low carbohydrate intake, ketogenesis is promoted as a source of energy [5, 6]. Ketogenesis includes overproduction of acetoacetate, beta-hydroxybutyric acid, and acetone, with these processes occurring mainly in the liver. Ketogenesis associated with low carbohydrate diets or dietary ketosis is thought to be safe, which is different from pathologic cases of ketoacidosis that happens in patients with diabetes [5, 6]. During normal and controlled conditions of caloric intake and hydration, use of very low carbohydrate diet seems to produce safe levels of ketosis [13–15]. Studies have shown that restricting carbohydrate intake is safe from an electrolyte and acid base stand point, especially with adequate caloric and fluid intake as performed in such studies [13–15]. Nonetheless, there are several cases in the literature of severe acidosis associated with ketogenic diets [16–21]. Acute kidney injury, dehydration, and other complications have been also reported in the pediatric population adhering to ketogenic diets for epilepsy management [22, 23]. Ingestion of dietary forms of vinegar is usually safe. Dietary vinegar contains acetic acid [24, 25]. Normally, acetic acid is metabolized to bicarbonate in the liver and should not alter acid base balance; however, there may be a delay between the actual ingestion and buffering which could create a temporary acid base disturbance [26]. Vinegar may be ketogenic as well [27]. A link between acetic acid consumption and ketosis has been demonstrated in animal studies. [28] Reports of complications or fatalities related to vinegar or acetic acid ingestion are mostly secondary to consumption of concentrated forms of acetic acid [29–31]. We did find one fatality reported to homicidal massive ingestion of dietary vinegar in a pediatric patient [32]. The combination of metformin to carbohydrate-restricted diets has been reported to aid in management of diabetes patients [33]. None the less, metformin may exacerbate ketosis through inhibition of gluconeogenesis and stimulation of fatty acid oxidation as previously reported [34]. Our case is unique in a way that the patient engaged in aggressive carbohydrate restriction along with frequent ingestion of vinegar in the setting of an increasing dose of metformin, and all these factors worked in concert to promote unrestricted ketogenesis which led to life-threatening anion gap acidosis. She was not following with a dietician or a healthcare professional to guide her dietary changes and did not maintain appropriate fluid intake. To our knowledge, this is the first case in literature that is reported with severe acidosis in setting of ketogenic diet, vinegar ingestion, and metformin use. This should serve as a good resource for clinicians and public to educate about the risks of various, presumed to be harmless, dietary changes. In conclusion, ketogenic diets and vinegar consumption can lead to life-threatening acidosis. It is imperative that trained healthcare providers supervise patients seeking weight loss if they want to engage in either of these options to avoid potentially serious complications. We should also work in our communities to educate the public and produce reliable media outlets to help spreading reliable medical advice.

## Figures and Tables

**Table 1:** Laboratory investigations at time of admission and discharge.

Laboratory variables (units)	Labs on admission	Labs on discharge	Normal values
PCO_2_ (mmHg)	31	43	41–51
HCO_3_ (mmol/L)	10.5	23.5	23–29
pH	7.15	7.35	7.31–7.41
WBC (×10^9^/L)	10.6	2.0	4.5–10
Hemoglobin (g/dl)	11.4	7.9	12–16
Platelets (×10^9^/L)	231	93	150–440
Sodium (meq/L)	131	142	135–145
Potassium (meq/L)	3.8	4.1	3.5–5.0
Chloride (meq/L)	94	104	98–108
Blood sugar (mg/dl)	108	103	74–100
HbA1C (%)	5.1	NA	<5.7
Osmolality (mOsm/kg)	298	300	281–297
Anion gap	27	6	5–14
Serum albumin	4.7	3.0	3.1–4.5
Serum acetone	3+	NA	Negative
BUN (mg/dL)	19	6.1	7–18
Creatinine (mg/dL)	2.37	0.9	0.6–1.10
Serum ethanol (mg/dL)	18	NA	Negative
Lactic acid (mmol/L)	4.19	1.89	0.7–2.10
Salicylate level (mg/dL)	<2	NA	2–20
Methanol level	Negative	NA	Negative
D-Lactate	<0.2	NA	<0.50
Isopropyl ethanol	Negative	NA	Negative
Antipancreatic islet cell Ab	Negative	NA	Negative

NA, values not available.

## Data Availability

The results and findings pertinent to this case report are included within the article.

## Abbreviations

AKI: Acute kidney injury
SGLT2: Sodium-glucose cotransporter-2
D5W: Dextrose 5% in water.

## References

[1] Hales C. M., Carroll M. D., Fryar C. D., Ogden C. L. (2017). Prevalence of obesity among adults and youth: United States, 2015–2016. *NCHS Data Brief*.

[2] Hruby A., Hu F. B. (2015). The epidemiology of obesity: a big picture. *Pharmacoeconomics*.

[3] Kruger J., Galuska D. A., Serdula M. K., Jones D. A. (2004). Attempting to lose weight. *American Journal of Preventive Medicine*.

[4] Nicklas J. M., Huskey K. W., Davis R. B., Wee C. C. (2012). Successful weight loss among obese U.S. adults. *American Journal of Preventive Medicine*.

[5] Paoli A. (2014). Ketogenic diet for obesity: friend or foe?. *International Journal of Environmental Research and Public Health*.

[6] Paoli A., Rubini A., Volek J. S., Grimaldi K. A. (2013). Beyond weight loss: a review of the therapeutic uses of very-low-carbohydrate (ketogenic) diets. *European Journal of Clinical Nutrition*.

[7] Kondo T., Kishi M., Fushimi T., Ugajin S., Kaga T. (2009). Vinegar intake reduces body weight, body fat mass, and serum triglyceride levels in obese Japanese subjects. *Bioscience, Biotechnology, and Biochemistry*.

[8] Jane M., Hagger M., Foster J., Ho S., Pal S. (2018). Social media for health promotion and weight management: a critical debate. *BMC Public Health*.

[9] Pirraglia P. A., Kravitz R. L. (2013). Social media: new opportunities, new ethical concerns. *Journal of General Internal Medicine*.

[10] Korownyk C., Kolber M. R., McCormack J. (2014). Televised medical talk shows—what they recommend and the evidence to support their recommendations: a prospective observational study. *BMJ*.

[11] Churuangsuk C., Kherouf M., Combet E., Lean M. (2018). Low-carbohydrate diets for overweight and obesity: a systematic review of the systematic reviews. *Obesity Reviews*.

[12] Masood W., Uppaluri K. R. (2020). *Ketogenic Diet*.

[13] Yancy W. S., Olsen M. K., Dudley T., Westman E. C. (2007). Acid-base analysis of individuals following two weight loss diets. *European Journal of Clinical Nutrition*.

[14] Phinney S. D., Bistrian B. R., Wolfe R. R., Blackburn G. L. (1983). The human metabolic response to chronic ketosis without caloric restriction: physical and biochemical adaptation. *Metabolism*.

[15] Gomez-Arbelaez D., Crujeiras A. B., Castro A. I. (2017). Acid-base safety during the course of a very low-calorie-ketogenic diet. *Endocrine*.

[16] Freeman T. F., Willis B., Krywko D. M. (2014). Acute intractable vomiting and severe ketoacidosis secondary to the dukan diet. *The Journal of Emergency Medicine*.

[17] Chalasani S., Fischer J. (2008). South Beach Diet associated ketoacidosis: a case report. *Journal of Medical Case Reports*.

[18] Basnet S., Tachamo N., Nazir S., Dhital R., Jehangir A., Donato A. (2019). Severe anion gap metabolic acidosis associated with initiation of a very low-carbohydrate diet. *Journal of Community Hospital Internal Medicine Perspectives*.

[19] Shah P., Isley W. L. (2006). Ketoacidosis during a low-carbohydrate diet. *New England Journal of Medicine*.

[20] Chen T.-Y., Smith W., Rosenstock J. L., Lessnau K.-D. (2006). A life-threatening complication of Atkins diet. *The Lancet*.

[21] von Geijer L., Ekelund M. (2015). Ketoacidosis associated with low-carbohydrate diet in a non-diabetic lactating woman: a case report. *Journal of Medical Case Reports*.

[22] Yoo K. H., Yim H. E. (2019). Acute tubular necrosis associated with the ketogenic diet in a child with intractable epilepsy. *Childhood Kidney Diseases*.

[23] Kang H. C., Chung D. E., Kim D. W., Kim H. D. (2004). Early- and late-onset complications of the ketogenic diet for intractable epilepsy. *Epilepsia*.

[24] Johnston C. S., Gaas C. A. (2006). Vinegar: medicinal uses and antiglycemic effect. *MedGenMed: Medscape General Medicine*.

[25] US Food and Drug Administration (2014). *CPG Sec. 525.825 Vinegar, Definitions—Adulteration with Vinegar Eels*.

[26] Lhotta K., Höfle G., Gasser R., Finkenstedt G. (1998). Hypokalemia, hyperreninemia and osteoporosis in a patient ingesting large amounts of cider vinegar. *Nephron*.

[27] Harvey C. J. d. C., Schofield G. M., Williden M. (2018). The use of nutritional supplements to induce ketosis and reduce symptoms associated with keto-induction: a narrative review. *PeerJ*.

[28] Eaton M., MacKay R. H. B., Carne H. O., Arne N. (1940). Wick *ketogenic Activity of acetic acid*. *Journal of Biological Chemistry*.

[29] Tibballs J., Cathie R., Buist M., Shimizu K., Stokes K., Millar J. (2006). Upper airway obstruction caused by ingestion of concentrated acetic acid. *Anaesthesia and Intensive Care*.

[30] Jurim O., Gross E., Nates J., Eldor A. (1993). Disseminated intravascular coagulopathy caused by acetic acid ingestion. *Acta Haematologica*.

[31] Ratcliffe A., Baker A., Smith D. (2018). Successful management of 70% acetic acid ingestion on the intensive care unit: a case report. *Journal of the Intensive Care Society*.

[32] Shields L. B. E., Rolf C. M., Hunsaker J. C. (2016). Sudden death due to forced ingestion of vinegar. *Forensic Science International*.

[33] Müller J. E., Sträter-Müller D., Marks H.-J. (2011). Carbohydrate restricted diet in conjunction with metformin and liraglutide is an effective treatment in patients with deteriorated type 2 diabetes mellitus: proof-of-concept study. *Nutrition & Metabolism*.

[34] Schwetz V., Eisner F., Schilcher G. (2017). Combined metformin-associated lactic acidosis and euglycemic ketoacidosis. *Wiener klinische Wochenschrift*.

